# National survey of clinical communication assessment in medical education in the United Kingdom (UK)

**DOI:** 10.1186/1472-6920-14-10

**Published:** 2014-01-13

**Authors:** Anita Laidlaw, Helen Salisbury, Eva M Doherty, Connie Wiskin

**Affiliations:** 1 Medical School, University of St Andrews, Medical and Biological Sciences building, North Haugh, St Andrews, Fife KY16 9TF, Scotland; 2 Department of Primary Care Health Sciences, University of Oxford, Oxford, England; 3 National Surgical Training Centre, The Royal College of Surgeons in Ireland, Dublin, Ireland; 4 College of Medical and Dental Sciences, University of Birmingham, Birmingham, England

**Keywords:** Clinical communication, Assessment, Survey

## Abstract

**Background:**

All medical schools in the UK are required to be able to provide evidence of competence in clinical communication in their graduates. This is usually provided by summative assessment of clinical communication, but there is considerable variation in how this is carried out. This study aimed to gain insight into the current assessment of clinical communication in UK medical schools.

**Methods:**

The survey was sent via e-mail to communication leads who then were asked to consult with all staff within their medical school involved in the assessment of communication.

**Results:**

Results were obtained from 27 out of 33 schools (response rate 82%) and a total of 34 courses. The average number of assessments per year was 2.4 (minimum 0, maximum 10). The Objective Structured Clinical Exam (OSCE) was the most commonly used method of assessment (53%). Other assessments included MCQ and workplace based assessments. Only nine courses used a single method of assessment. Issues raised included, logistics and costs of assessing mainly by OSCE, the robustness and reliability of such exams and integration with other clinical skills.

**Conclusions:**

It is encouraging that a variety of assessment methods are being used within UK medical schools and that these methods target different components of clinical communication skills acquisition.

## Background

The ability to communicate is recognised to be one of the key components of effective medical practice. In the United Kingdom (UK) the General Medical Councils (GMC) Tomorrows’ Doctors 2009 [1] outlines several competency outcomes relating to clinical communication. Graduates should be able to: ‘Communicate effectively with patients and colleagues in a medical context’. This should include skills such as; clear, sensitive and effective communication with not only patients, their relatives or other carers, but also colleagues, that the efficacy of communication should not depend on the age, social, cultural or ethnic backgrounds, disabilities of an individual, nor the media by which the communication is delivered. Finally, that the communication occurring should be effective within any healthcare context, including with vulnerable patients and not depend on the role an individual is fulfilling [1]. A consensus statement has also been published by the UK Council of Clinical Communication in Undergraduate Medical Education [2] which describes the suggested clinical communication curriculum content for undergraduate medical education in the UK, this covers similar areas to the GMC Tomorrow’s Doctors guidelines [1] but in greater depth. There is therefore guidance for UK medical schools on what areas of clinical communication to include within their courses. Similar guidelines are in place for other countries, for example the Australian Medical Council states that graduates should be competent in: ‘communication skills, including being able to listen and respond, and to convey information clearly, considerately and sensitively to patients and their families, doctors, nurses, other health professionals and the general public.’ (page 2) [3], whilst in the USA doctors should have the ability to …..‘communicate effectively, both orally, and in writing, with patients, patients’ families, colleagues, and others who physicians must exchange information in carrying out their responsibilities.’ (page 7) [4].

In order to ensure that graduates are indeed competent in these skills, medical schools need to provide evidence of skill attainment which is often demonstrated via some form of assessment. George Miller developed a model of assessment of clinical skills competence and performance which describes different aspects of skill acquisition [5], from acquiring theoretical knowledge on what the skills are (described in Millers model as ‘knows’), knowledge of how to apply these skills (described as ‘knows how’), being able to competently carry out the skills on specific occasions (described as ‘shows how’), through to competently carrying out the skills on a day to day basis (described in Millers model as ‘does’). In relation to clinical communication, there is good evidence that the components of ‘shows how’ and ‘does’ are closely related, and that scores on assessments in medical school are correlated with workplace assessment of the same skills [6]. The evidence for an association between other components, for example the knowledge ‘knows’ and ‘shows how’ is less clear, with some studies showing no association [7] and others showing negative associations later on in training [8]. George Miller himself stated that ‘no single assessment method can provide all the data required for anything so complex as the delivery of professional services by a successful physician’ [5]. An assessment of all aspects of clinical communication, including knowledge, understanding, skills and performance on a day to day basis should be the gold standard. There is evidence that assessment of knowledge and skills competence is a better predictor of clinical performance than skills competence testing on its own [9]. It is not known whether a breadth of assessment methods are currently being used in UK medical schools, or whether there is reliance on one method over another.

The aim of this study was to provide a clear picture of the current summative assessment of clinical communication knowledge and practice in UK medical schools. We seek to answer three main questions; 1) How often is clinical communication and/or the knowledge base of clinical communication assessed?, 2) When within the students progression through the course is clinical communication and/or its’ knowledge base assessed?, 3) What methods are used to assess clinical communication and/or its’ knowledge base?

## Methods

### Data collection

The survey was generated via a subgroup of the UK Council of Clinical Communication Teaching in Undergraduate Medical Education (UK Council). The UK Council consists of leads for clinical communication teaching from each Medical School in the UK. A first draft of the survey was considered by a meeting of this group who commented on content and format and a revised survey was developed (see Additional file 1).

The questionnaire was then sent to the leads for clinical communication teaching in all of the UK medical Schools via e-mail. The clinical communication lead for each school was asked to complete it for all summative assessment after consultation with others involved in clinical communication assessment in their school. As the majority of the leads for clinical communication for each Medical School had been active participants in the development of the survey, there was a willingness to participate in this evaluation of assessment and an appreciation of the importance of determining all existing assessment opportunities within each curriculum. Participants were initially sent out the survey during May 2009 via e-mail. Two e-mail reminders were sent to those who had not submitted a response and the survey was completed by December 2009.

Once data was collected from each school they were merged to create a database.

### Description of questionnaire

The questionnaire asked schools to list all occurrences of clinical communication assessment, recording when they occurred, the type of assessment, the context and, if it was a practical assessment, who was involved and the type of scale used to assess. Schools were also asked to provide open responses to various questions including ‘What is the greatest challenge in the assessment of communication in your medical school?’. For full questionnaire see Additional file 1.

### Data analysis

For graphical display, some categories of assessment types or examiner types were merged for simplification. For example, the assessment type OSCE (Objective Structured Clinical Examination) category here includes OSCEs using simulated and real patients whilst the assessment type category of workplace based assessment includes workplace assessment and mini-CEX (mini clinical evaluation exercise). Summary data was generated using Microsoft Excel 2010 and any statistical analysis was carried out in SPSS v19. Data was examined to determine differences in assessment between years of study (one way ANOVA) and curricula types (Fisher’s exact test). Free text responses were grouped thematically by one author (HS) and the content summarised following face to face discussion with another author (AL).

After consultation with the Convenor of the St Andrews Medical School Teaching and Research Ethics Committee ethical permission was not sought for this initiative as it was considered an internal UK Council of Clinical Communication in Undergraduate Medical Education audit of assessment practice within schools to gain a clear picture of current practice and to allow the informed consideration of developing national standards.

## Results

### Courses summary information

Responses were collected between May and December 2009. Twenty seven out of 33 schools submitted responses, a response rate of 82%. These 27 medical schools offered 34 separate courses, including 8 postgraduate entry (PG = 24%). Clinical communication leads for each school were responsible for self-reporting curriculum types and entry levels (entry level = both when PG are recruited onto the same course as undergraduates (UG)). Summary information of the courses is available in Table 1.

**Table 1:** Summary information of the medical courses participating in the study

**School ID**	**Course ID**	**Entry**	**Duration (years)**	**Course type**	**Cohort**	**Long case**	**MCQ SWA**	**Workplace based**	**OSCE**	**Other**	**Portfolio/reflection**	**Presentation**	**Written**	**Total assessments**
1	2	Both	5	Integrated	180	0	0	0	8	0	0	0	0	8
2	23	UG	5	PBL	350	0	0	0	5	1	0	0	0	6
	24	PG	4	PBL	60	0	0	0	4	1	0	0	0	5
3	18	UG	5	Other	400	1	0	3	11	1	1	2	5	21
	19	PG	4	PBL	60	1	0	1	13	1	1	1	1	18
4	30	Both	5	Integrated	130	0	2	1	4	0	3	0	2	12
5	21	Both	5	Integrated	250	0	0	0	5	2	0	0	0	7
6	5	UG	6	Traditional	290/130	0	0	0	4	0	3	0	0	4
	6	PG	4	Other	24	0	0	0	4	0	0	0	0	4
7	1	UG	5	Traditional	300	0	0	0	5	0	0	0	0	5
8	22	Both	5	Integrated	150	0	0	3	5	3	4	0	0	15
9	7	Both	2	Integrated	102	0	4	0	2	2	4	0	3	15
10	8	Both	5	Integrated	280	1	0	1	4	1	2	0	0	8
11	31	UG	5	Integrated	240	0	0	0	6	0	0	0	0	6
12	29	Both	5	PBL	130	2	0	0	3	2	0	0	0	7
13	34	UG	6	Integrated	300	0	3	5	8	0	2	0	0	13
	35	PG	4	Integrated	50	0	1	3	8	0	2	0	0	11
14	9	Both	5	Integrated	120	0	0	2	10	0	0	0	0	12
15	10	UG	5	PBL	330	0	0	0	10	0	2	0	0	12
16	11	UG	5	PBL	450	0	6	2	12	1	6	0	3	30
17	12	Both	Other	Integrated	360	0	3	2	6	0	2	1	0	14
18	32	UG	5	Traditional	260	1	0	3	4	2	3	1	0	11
	33	PG	4	PBL	90	0	0	3	4	0	0	0	0	4
19	15	UG	6	Traditional	130	0	0	2	6	2	0	0	1	11
	16	PG	4	Integrated	28	0	0	0	4	0	0	0	0	4
20	28	UG	5	PBL	220	0	0	2	2	2	5	0	8	17
21	25	Both	4	Integrated	280	0	0	0	0	5	0	0	0	5
22	27	UG	5	Integrated	250	1	0	5	5	1	0	3	2	12
23	13	UG	3	Integrated	160	0	0	0	6	0	0	0	0	6
24	3	PG	4	PBL	100	0	0	0	5	0	0	0	0	5
	4	UG	5	Traditional	200	0	0	0	7	0	0	0	0	7
25	17	Both	6	Traditional	380	0	4	0	3	0	0	0	0	7
26	14	Both	5	Integrated	150	0	0	0	5	0	0	0	0	5
27	20	PG	4	Other	178	5	0	0	3	3	0	0	0	11

Entry level (postgraduate (PG), undergraduate (UG) or both), duration in years, the self-reported course types (integrated, problem-based learning, traditional or other) and the cohort size is reported. A detailed breakdown of methods used to assess clinical communication for each course is also included.

### How often and when is clinical communication assessed?

**Figure 1:**
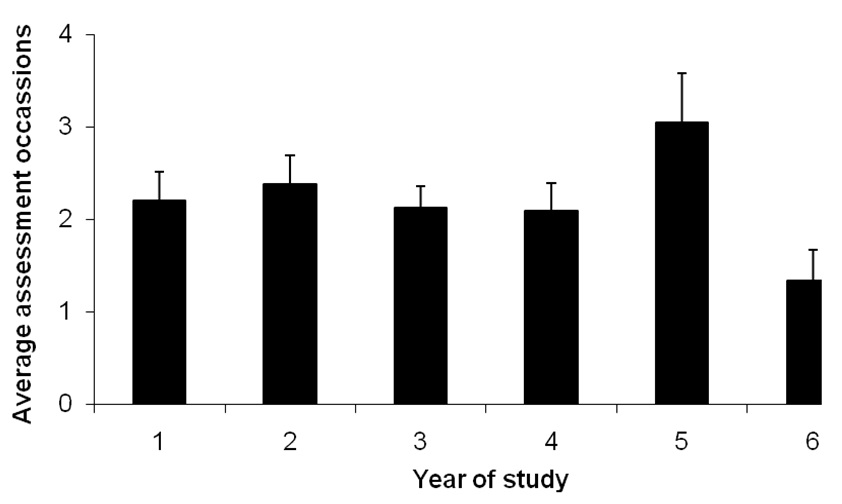
**Average assessments of clinical communication per year.** The average number of assessments reported in each year for all courses is reported. Error bars represent standard errors.

The average total number of occasions for assessing clinical communication on all courses was 10.8 ± 2.7 (standard error), with a minimum of 4 and a maximum of 30. When this was investigated taking the length of the course into account, the average number of assessments per year was 2.4 ± 0.26 (minimum = 0, maximum = 10). Figure 1 shows the average number of assessments per year and it is clear there is a peak is assessment in year five, representing finals (although this difference did not reach statistical significance). There was no significant difference in the total number of assessments of clinical communication between different curricula types.

### Methods of assessing clinical communication

**Figure 2:**
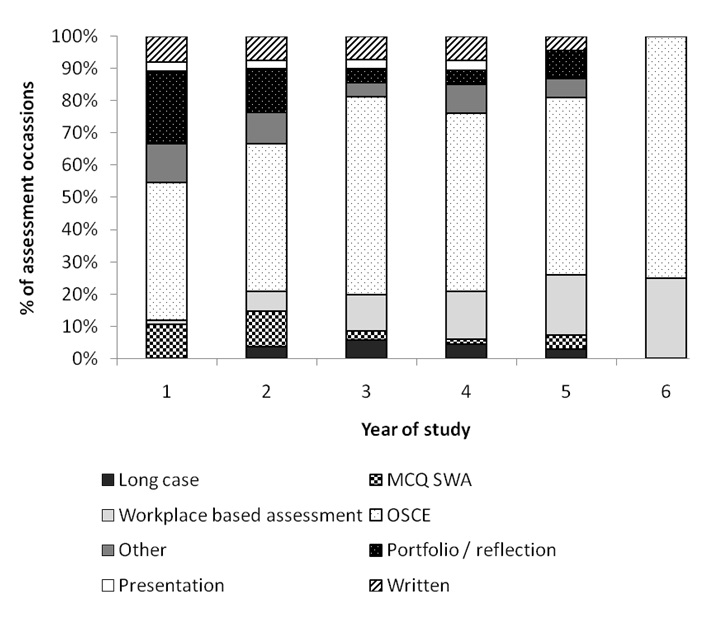
**Assessment type (%) by year of study for all curricula.** The % distribution of the variety of assessment types for each year of study for all assessment occasions reported in this survey.

There are numerous methods of assessing clinical communication, Table 1 and Figure 2 show the different ways that clinical communication is currently assessed in medical courses within the UK. It can be seen from both Table 1 and Figure 2 that the OSCE style examination is the most commonly used method of assessing clinical communication in UK medical schools. Overall, the OSCE is used in 53% of assessment occasions. The average number of different types of assessments used on a course to assess clinical communication was 3.12 ± 0.34 (min = 1, max = 7). Only nine courses (26%) assessed clinical communication by one type of assessment. There was little variation between curriculum types. An interesting trend was observed in type of assessment with progression through a course. MCQ (multiple choice questions), SWA (short written answers) and portfolio assessments occur in the early years, OSCE assessments throughout, and workplace based assessments occur more often in years four to six.

We examined where assessments were taking place within teaching with respondents being asked to state the context of the assessment. Assessment often occurred in end of year or end of semester exams or within specialty blocks. Within the specialty blocks, where stated, general practice, mental health, paediatrics and obstetrics and gynaecology stood out as commonly containing clinical communication assessment.

### OSCE type assessments

As OSCE type assessments were the most commonly used, this method of assessment was examined more closely. Eighty percent of OSCE type assessments used simulated patients or actors with only 20% using real patients. Seventy four percent of examiners assessing during an OSCE were health professionals. We examined this further by specifically asking whether health professionals used for assessing clinical communication were experts in the field of communication: 63% were experts, whilst 37% were not. Eleven percent of examiners were simulated patients and 5% were non-health professional communication tutors. Interestingly 3% of OSCE examiners were peers, whilst 7% were classified as ‘other’.

We also enquired about the type of assessment tools used during OSCE type examinations. In 74% of OSCE examinations the assessment tool was a combination of checklist and global rating scale. In only 10.5% of OSCE type examinations, a checklist was the only means of assessing the candidate, while in the remaining occasions (15.5%) only a global rating of the candidates’ competence was used. The number of OSCE type assessments a student experiences as they progress through each year remains fairly level, at around 2, with only a slight rise in year 5, to 2.9 ± 0.29.

### What is the greatest challenge in the assessment of communication in your medical school?

The main challenges in assessment of communication skills identified by the respondents were grouped under four headings: logistics, standard setting and validity, faculty development and integration of content and process.

There are significant logistical problems around examining large numbers of students in a one-to-one OSCE style examination, in terms of time, cost of simulated patients and examiners and availability of rooms. Lack of resources was seen as a major challenge in several of the schools.

Defining different levels of competence at the different stages of the course was also reported as a significant challenge; this appeared to be a particular problem for assessments in the early stages of training. Concerns about the robustness and validity of assessments were expressed by several schools, and these concerns were often associated with dissatisfaction with the number of assessments because of logistical problems. Robustness is also related to examiner expertise and training and ensuring consistency across examiners was mentioned as a problem by several schools. One respondent commented: “I feel some of the students communicate better than some of the non-specialist examiners used in the OSCEs, so an examiner may not always recognise excellent skills used by students and therefore award inappropriate marks.”

The degree of integration of medical content with communication process within an assessment was also reported to be a challenge.

## Discussion

The results of this survey have provided a clear picture of clinical communication assessment in undergraduate medical curricula in the UK. The number of summative assessments of clinical communication was fairly stable with schools assessing students an average of twice a year, with a peak in assessment occurring in year five associated with finals. An average of two assessments per year may raise issues of reliability, but as the nature of these assessments varied so widely it is not possible to generalise on this point. Some assessments were multi-station OSCE exams whereas others may be single station or written answer. Various studies have investigated the issue of generalisability in the past with the numbers of individual measures of skills required to reliably assess the competence of an individual ranging from seven [10] to 14 [11]. In addition, formative assessment, which lay outside the scope of this questionnaire, may provide other opportunities for picking up students with poor performance in this area. The consequences of failing one of the assessments reported here are discussed in another paper [12], which highlights that for some students these examinations are high stake, whilst for others there are few consequences. Thus there is an imperative for at least some of these assessments to ensure reliability.

This study has demonstrated that UK medical schools assess clinical communication throughout their curricula in a variety of different ways. The practical assessment of competence, the OSCE, has become the most common form of assessment (at 53% of all assessment occasions).

The OSCE was initially described by Harden et al. in the 1970’s [13] and has since gained popularity. The OSCE, in its most common form, measures only one aspect of clinical communication from Miller’s pyramid model of assessment, the ‘shows how’ [5] component. If medical schools were solely utilising this method of assessment they could be missing out on testing the other components of skills acquisition. However, this study has identified that UK medical schools use on average three different methods of assessment, including portfolios, multiple choice or short written answer questions and workplace based assessment. It is encouraging that such variety of assessment is used by the majority of schools, with only nine courses relying on one method of assessment. OSCE assessment has little correlation with assessments of knowledge, verbal competence, or written communication [8,14,15], thus to ensure rounded assessment, several methods would be required.

An interesting pattern of assessment method usage was observed. OSCE style exams were common throughout a student’s progression through medical school, but knowledge assessment (‘knows’) was more common in the early years (via multiple choice and short written answer questions) along with understanding how to apply that knowledge (‘knows how’, through portfolio) whilst performance (‘does’) tended to be assessed in later, more clinical, years via workplace based methods. This may follow the pattern of most of the students’ learning in other areas of the curriculum from theoretical, knowledge-based to practical, skills-based.

Most assessments of clinical communication occurred within end of year or end of semester examination periods or at the end of specialty blocks. Across the specialties (where stated) general practice, mental health, paediatrics and obstetrics and gynaecology were the most likely to have assessments of clinical communication within them. In a UK survey which included the context of clinical communication training, these specialties were predominant so it is perhaps unsurprising that this is also often the context of assessment [16]. It is thought that these specialties in particular emphasise and include the doctor-patient relationship as key to their clinical practice [17].

A further interesting point uncovered in this study was the use of actors in clinical communication assessment. For the OSCE style exams, 80% involved the use of an actor playing the role of a patient. This implies a considerable expense, as indeed does any practical type assessment.

Finally, this study considered the issue of examiners. There is some evidence that the actual participants of an interaction are better placed to judge the appropriateness of the communication occurring than an impartial observer [18], but there is conflicting evidence of the correlation between the ratings given by simulated patients and expert examiners [19-22]. This study shows that in 74% of OSCE style assessments health professionals were the examiners, with simulated patients contributing in 11% of cases. Further research is required to investigate in what way the judgement of students’ skills by simulated patients differs from those of other examiners and whether their contribution would increase the reliability or validity of these assessments.

Assessing clinical communication was reported to involve several challenges by respondents, and in particular integration with clinical content was highlighted. In schools that teach communication in the early years of the course, the assessment of these skills in the absence of sound clinical knowledge can be difficult. More knowledgeable students examined in later years in designated communication stations tend to focus on the process of interaction rather than completing the clinical task. Conversely, if communication is examined in an integrated fashion, which many see as preferable, it then may be difficult to unpick the communication from the other clinical skills and knowledge demonstrated, this has been raised as a concern in the UK [12]. This last point may be less of a problem than it appears as there is literature to show that poor communicators are generally poor in a range of domains and other in course assessments may identify these students [23].

This study has several limitations. Although an 82% response rate was achieved this is still not a comprehensive report of the clinical communication assessment occurring within UK medical schools. However, it does provide a snap shot of the assessment practices in this area in the majority of schools.

The leads for clinical communication in each school were asked to complete the questionnaire and their knowledge of all assessment occasions may not have been complete. In particular, there may be an underreporting of assessments in some specialty blocks from which responses were not received. In addition, as mentioned above, in the later years of many courses communication may be regarded as an integrated skill. Most medical examiners would assume they were marking communication as a skill inherent in the medical interview and would object to the isolation of communication when marking an OSCE involving a consultation. Thus our survey probably under represents the number of assessments of clinical communication.

## Conclusions

This study is the most complete survey of clinical communication assessment within undergraduate medical education in the UK to date. Medical students appear to have their clinical communication assessed on average two times a year, and, although the OSCE is the most common form of assessment schools use, it is encouraging that a variety of assessment methods are being used and that these methods target the different components of clinical communication skills acquisition.

## Competing interests

The authors report no declarations of interest.

## Authors’ contributions

AL: Contributed to revisions of the draft survey, involved in collecting results (main contact). Analysed results, was the main author of the manuscript. HS: Contributed to revisions of the draft survey, involved in collecting results. Involved in writing of the manuscript. ED: Contributed to revisions of the draft survey, involved in collecting results and commented on drafts of the manuscript. CW: Contributed to revisions of the draft survey, involved in collecting results and commented on drafts of the manuscript. All authors read and approved the final manuscript.

## Authors’ information

Anita Laidlaw is a Principal Teaching Fellow and Convenor of communication skills at the Medical School, University of St Andrews, UK. Her current research interests are psychological and cognitive factors affecting communication and pedagogy.

Helen Salisbury is a GP and Honorary Senior Clinical Lecturer in the Department of Primary Care Health Sciences and Oxford University where she is medical advisor to the Health Experiences Research Group. Her current interests include the role of individual patient experience in medical education.

Eva Doherty is Senior Lecturer/Clinical Psychologist at the Royal College of Surgeons in Ireland (RCSI). She is Director of the Human Factors and Patient Safety teaching and research programme at the National Surgical Training Centre in RCSI. Current research interests include personality assessment in medical education, emotional intelligence measurement and the assessment of Human Factors training programmes.

Connie Wiskin is a Senior Lecturer at the College of Medical and Dental Sciences, University of Birmingham. Her research specialties are interactive assessment and educational evaluation. She is Academic Lead for the Birmingham Elective, and Co-Director of the Interactive Studies Unit.

## Pre-publication history

The pre-publication history for this paper can be accessed here:

http://www.biomedcentral.com/1472-6920/14/10/prepub

## Supplementary Material

Additional file 1 UK Council of Clinical Communication in Undergraduate Medical Education Assessment Survey. Click here for file
