# Supplementary material for: National survey of clinical communication assessment in medical education in the United Kingdom (UK)
Source: BMC Med Educ. 2014 Jan 13;14:10. doi: 10.1186/1472-6920-14-10 (PMC3898466; doi:10.1186/1472-6920-14-10)
Supplement: Additional file 1 — UK Council of Clinical Communication in Undergraduate Medical Education Assessment Survey. [file 1472-6920-14-10-S1.pdf]

UK Council of Clinical Communication in Undergraduate Medical Education

There are two parts to this survey: 1) Your institution and views and 2) Assessment occasions

1) Your institution and views

This part should be completed by the lead for clinical communication within your institution. Please complete each question. Some questions contain drop down menus for you to select an option (questions 5 - 7). For the open response questions simply type in your response (questions 8 - 12). If your institution runs two medical courses, e.g. undergraduate and postgraduate entry courses which are separate, please complete a 'Your institution and views' worksheet for each course. If responses to the open questions are the same for this second course, you do not need to repeat the answer.

1. Name of School
2. Your name
3. Your e-mail
4. Number of students per year
5. Course structure
6. Duration
7. Entry Qualifications
8. What happens to students who fail compulsory communication assessments?
9. Are there any compulsory communication assessments, failure of which would prevent progression? If so which and can you describe it?
10. What support is available for students failing communication assessments?
11. Are poor standards of communication identified by any other processes in your institution (e.g. fitness to practice)?
12. What is the greatest challenge in the assessment of communication in your medical school?

2) Assessment occasions

This section should be compiled by the lead for clinical communication within your institution. This sheet should list and describe all occasions where communication is compulsorily assessed on each course. Again, complete two worksheets if two separate courses are run. Information may have to be sought from others involved in assessing within the institution to complete this part of the survey e.g within specialty modules. If this is the case simply forward a copy of this attachment to them which they can complete and return to you. Ideally we'd prefer it if you could copy the replies you receive into a single 'Assessment occasions' spread sheet to return.

When and how is communication compulsorily assessed for all students?

Occasion	Year	Context e.g specialty	Summative or Formative	Method	By whom	For practical assessment are mark sheets:
				• OSCE type (actor / simulated patient)<br>• OSCE type (real patient)<br>• Long case<br>• Mini-CEX<br>• Workplace<br>• Portfolio/ Reflection<br>• Written communication (reports / charts)<br>• Written (SWA/MCQ)	• Comm teacher: health professional<br>• Comm teacher: non-health professional<br>• Other health professional<br>• Actor / simulated patient<br>• Peer<br>• Other	• Global<br>• Checklist<br>• Both
